# Mechanisms Involved in Gut Microbiota Regulation of Skeletal Muscle

**DOI:** 10.1155/2022/2151191

**Published:** 2022-05-18

**Authors:** Guangyao Li, Binghui Jin, Zhe Fan

**Affiliations:** ^1^Department of General Surgery, The Third People's Hospital of Dalian, Dalian Medical University, Dalian, China; ^2^Department of Central Laboratory, The Third People's Hospital of Dalian, Dalian Medical University, Dalian, China

## Abstract

Skeletal muscle is one of the largest organs in the body and is essential for maintaining quality of life. Loss of skeletal muscle mass and function can lead to a range of adverse consequences. The gut microbiota can interact with skeletal muscle by regulating a variety of processes that affect host physiology, including inflammatory immunity, protein anabolism, energy, lipids, neuromuscular connectivity, oxidative stress, mitochondrial function, and endocrine and insulin resistance. It is proposed that the gut microbiota plays a role in the direction of skeletal muscle mass and work. Even though the notion of the gut microbiota–muscle axis (gut–muscle axis) has been postulated, its causal link is still unknown. The impact of the gut microbiota on skeletal muscle function and quality is described in detail in this review.

## 1. Introduction

Skeletal muscle is one of the largest organs, accounting for roughly half of the total body weight. Skeletal muscle produces heat, regulates blood sugar, storing amino acids, and alters the physiological characteristics of the body [1]. Skeletal muscle mass and function decline have been reported to affect 8%–13% of older adults [2], with clinical effects including frailty, loss of mobility, falls, fractures, disability, and increased mortality [3]. Numerous factors contribute to the loss of skeletal muscle mass and function, such as inflammatory states [4], age-related changes in the hormonal environment [5], insulin resistance [6], gut physiology [7], DNA damage, and mitochondrial dysfunction [8]. These mechanisms are enhanced in the presence of insufficient protein energy [9].

The physiological characteristics of skeletal muscle have been extensively studied in the past few decades, providing unique insights into the interconnection among organs [10]. As with the products secreted by skeletal muscle, external factors that may act on skeletal muscle can also play an important role in peripheral tissues. The gut microbiota has the potential to influence muscle function and quality [11]. The gut microbiota is increasingly being seen as a key factor in human wellbeing and disease, especially in older adults [12]. Although the gut microbiota is known for its role in nutrient absorption, it is closely associated with many other physiological processes [13]. Therefore, the interaction between the gut microbiota and human organs has become the focus of recent research [14].

Recent studies have demonstrated the existence of a gut microbiota-muscle axis, i.e., that muscle function and metabolism are largely dependent on the quantity and composition of the gut microbiota, and that the gut microbiota is expected to be a potential biological target for the prevention and treatment of muscle-related diseases such as sarcopenia and muscular dystrophy [15]. Furthermore, it is critical to clarify how the gut microbiota affects exercise load, modulates muscle function, and improves host fitness. The gut microbiota has a profound effect on skeletal muscle function and mass, and intervening in this axis may reverse the decline in skeletal muscle function and mass [13, 15–19]. This article reviews the progress of research on the effects of gut microbiota on the biological function of skeletal muscle and its mechanisms.

## 2. Gut Microbiota and Intestinal Barrier

### 2.1. Gut Microbiota

The human body consists of approximately 30 trillion cells that coexist with various microbial communities [20]. The human gut microbiome consists of 10–100 trillion microbes that are highly diverse, complex, constantly evolving, and colonize the digestive tract [21]. For host physiology, body homeostasis, and long-term health, functional interactions between gut microorganisms and hosts are critical. Although several studies have revealed how the gut microbiota impacts the liver and intestinal metabolism [22], there are few reports on how the gut microbiota regulates skeletal muscle, which is also one of the key metabolic organs [23]. The composition of the gut microbiome is influenced by a variety of factors, including genetics, age, diet, and exercise [24]. The human gut microbiota is dynamic throughout the life cycle, with the composition of gut microbes tending toward a steady state during the early years, but new research has found that the gut microbiota changes significantly in older adults (≥65 years) [25]. Antibiotics are known to cause changes in the microbiota composition, and older people are more inclined to use antibiotics more frequently [26], which may be one of the reasons for the changes in their gut microbiota composition.

To date, more than 9.9 million microbial genes have been found in human feces, with Bacteroides and Firmicutes accounting for the majority [27]. Probiotics are beneficial bacteria (e.g., *Lactobacillus*, *Bifidobacterium*, *Clostridium butyricum*, and *Bacillus subtilis*) [28]. Prebiotics are largely found in our gastrointestinal tract. Prebiotics are organic substances that the host cannot digest or absorb but which benefit the host's health. They feed beneficial bacteria and promote the growth and reproduction of beneficial bacteria [29]. The aging gut microbiota is highly characterized by a decrease in microbial diversity and beneficial bacteria, as well as a rearrangement of Bacteroides and Firmicutes, especially in older people, where individual differences in microorganisms can be greater [30, 31].

### 2.2. Intestinal Barrier

The intestinal tract of the organism has a relatively complete functional barrier, and intestinal barrier function refers to the function of the intestinal epithelium that can separate the intestinal lumen from the internal environment of the organism and prevent the invasion of pathogenic antigens. The normal intestinal barrier consists of mechanical barrier, chemical barrier, immune barrier, and biological barrier together [32].

The mechanical barrier is an intact intestinal mucosal epithelial structure closely connected to each other, which consists of a mucosal layer, intestinal epithelial cells, intercellular tight junctions, and submucosal lamina propria, and the intact intestinal mucosal epithelial cells and tight junctions between epithelial cells are the structural basis of the mechanical barrier [33]. Gastric acid, bile, various digestive enzymes, lysozyme, digestive juices, and antibacterial substances produced by parasitic bacteria in the intestinal lumen constitute the chemical barrier of the intestinal tract [34]. Stomach acid can destroy bacteria entering the gastrointestinal tract and inhibit bacterial adhesion and colonization of the gastrointestinal epithelium; lysozyme can destroy the cell wall of bacteria and cause bacterial lysis; digestive juices secreted by the intestine can dilute toxins and flush the intestinal lumen, making it difficult for potentially pathogenic bacteria to adhere to the intestinal epithelium [35, 36]. The immune barrier of the gut consists of immune cells, immune factors, and gut-associated lymphoid tissue. Immune cells initiate immune responses and form the intestinal mucosal immune system to protect the gut from external stimuli [36]. Immune factors enhance gut barrier function through immune rejection and bacterial clearance, in which immunoglobulin IgA plays an important role in regulating gut microbiota and maintaining immune homeostasis [37]. Gut-associated lymphoid tissue neutralizes antigenic substances by triggering local immune responses and can also secrete immunoglobulins to block the binding of bacteria to intestinal epithelial receptors, thereby effectively blocking the adhesion of harmful substances to the intestinal mucosa [38]. The normal parasitic flora in the intestine forms the biological barrier of the intestinal mucosa, and the metabolism of the gut microbiota can also regulate the mechanical, chemical, and immune barriers of the intestinal tract [39]. The biological barrier of the gut maintains the stability of the gut microbiota, and dysregulation of gut microbial homeostasis can lead to a decrease in beneficial microbes and an increase in harmful microbes, thereby compromising the health of the host [40].

Since birth, the microbiota has colonized the gastrointestinal tract and participates in many physiological processes in the host. Intestinal immune and endocrine function, energy homeostasis, and health are all influenced by the complex microbiota [41], which regulates inflammatory gene expression, innate immune effector cells (monocytes and macrophages), glucose tolerance, and gut hormone release, among other metabolic pathways [42, 43]. The gut microbiota and the gut barrier interact with each other. Intestinal cells regulate the composition of the gut microbiota by secreting antimicrobial peptides, and conversely, the gut microbiota can also affect the growth process of intestinal epithelial cells [34]. In mice, depletion of the gut microbiota compromises the intestinal epithelium, leading to altered patterns of microvillus formation and reduced cell renewal [44]. Probiotics form a biofilm to cover the intestinal mucosa, preventing the invasion of foreign bacteria, and they also produce acidic metabolites that lower the pH of the intestinal tract, thereby inhibiting the growth of harmful bacteria [45]. In addition, the accumulation of anaerobic bacteria and the invasion of exogenous pathogenic bacteria can lead to dysbiosis of the gut microbiota, damage the intestinal epithelial cells, and destroy the gut microbiota barrier [46].

### 2.3. Gut Microbiota Affects Skeletal Muscle Mass and Function

According to emerging evidence, the gut microbiota appears to play a role in regulating several muscle metabolic pathways [47]. Individual differences in gut microbiota relative abundance are linked to muscle mass and body weakness [48, 49], and higher gut microbiota diversity is linked to increased muscle mass [50]. In young women, the diversity of the gut microbiota is also related to skeletal muscle mass [51]. Increased numbers of *Oscillospira* and *Ruminococcus* and decreased numbers of Barnesellacae and Christensenellacea taxa are found in people with muscle wasting and physical weakness [48]. When compared to older people with low functional muscular strength, those with higher levels of *Prevotella*, *Barnesiella*, and *Barnesiella intestinihominis* have greater muscle strength [52]. *Barnesiella* and *Prevotella* have genes that produce short-chain fatty acids (SCFAs) [53].

Several studies from rodents have suggested that gut microbes may be related to the function and quality of skeletal muscle. The effects of gut microbiota shortage on skeletal muscle were studied in two animal investigations, which revealed that a lack of gut bacteria causes muscle mass loss [54, 55].The abundant Rikenellaceae group found in the gut microbiota of older mice is linked to a dose-dependent rise in muscular frailty index [56]. Higher *Sutterella* to *Barnesiella* ratio, altered inflammation and immune function, and decreased gastrocnemius and triceps size in rats with muscle atrophy were compared with healthy adult rats [47]. Comparison of germ-free (GF) mice lacking gut microbiota and pathogen-free (PF) mice with gut microbiota revealed skeletal muscle atrophy and decreased muscle mass in GF mice [23]. Ghrelin-deficient mice develop microbial dysbiosis at a young age and then lose muscle mass and function as they get older [57]. A decrease in gut bacteria can directly lead to muscle atrophy, according to two new studies [23, 54].

Antibiotics change the microbiota, and metronidazole has been shown to upregulate the expression of neurogenic atrophy-related proteins in skeletal muscle in earlier studies, as well as histone deacetylase 4, myostatin (MyoG), and FOXO1/FXOX3-mediated protein degradation, leading to skeletal muscle atrophy, thereby reducing muscle mass in the hind limb and muscle fiber volume in the tibialis anterior muscle of mice [58]. Similarly, antibiotic-treated mice resulted in muscle atrophy, reduced muscle mass, decreased running endurance, and increased *ex vivo* muscle fatigue [26, 59]. However, after inoculation with natural microbes in antibiotic-treated mice, the mice had increased muscle mass and a muscle mass/body weight ratio [59].

*In vitro* studies have also shown that gut microbial products can directly affect muscle mass [60]. The levels of two intestinal microbial metabolites (indoxyl sulfate and p-cresol sulfate) increase with age and play a vital part in muscle function [61]. Indoxyl sulfate, a biomarker of uremic sarcopenia, accelerates muscle atrophy by increasing inflammation levels, oxidative stress, and myasthenic gene expression and is negatively correlated with muscle strength and physical exercise [62]. Similarly, the gut microbiota that produces p-cresol sulfate, through insulin resistance and increasing muscle lipid content, ultimately contributes to poor muscle status [63]. Conversely, SCFAs are the end product of colonic protein fermentation and have many important physiological functions.

## 3. The Gut Microbiome Regulates Skeletal Muscle through a Variety of Mechanisms

### 3.1. Inflammation, Immunity, and Autophagy

**Figure 1:**
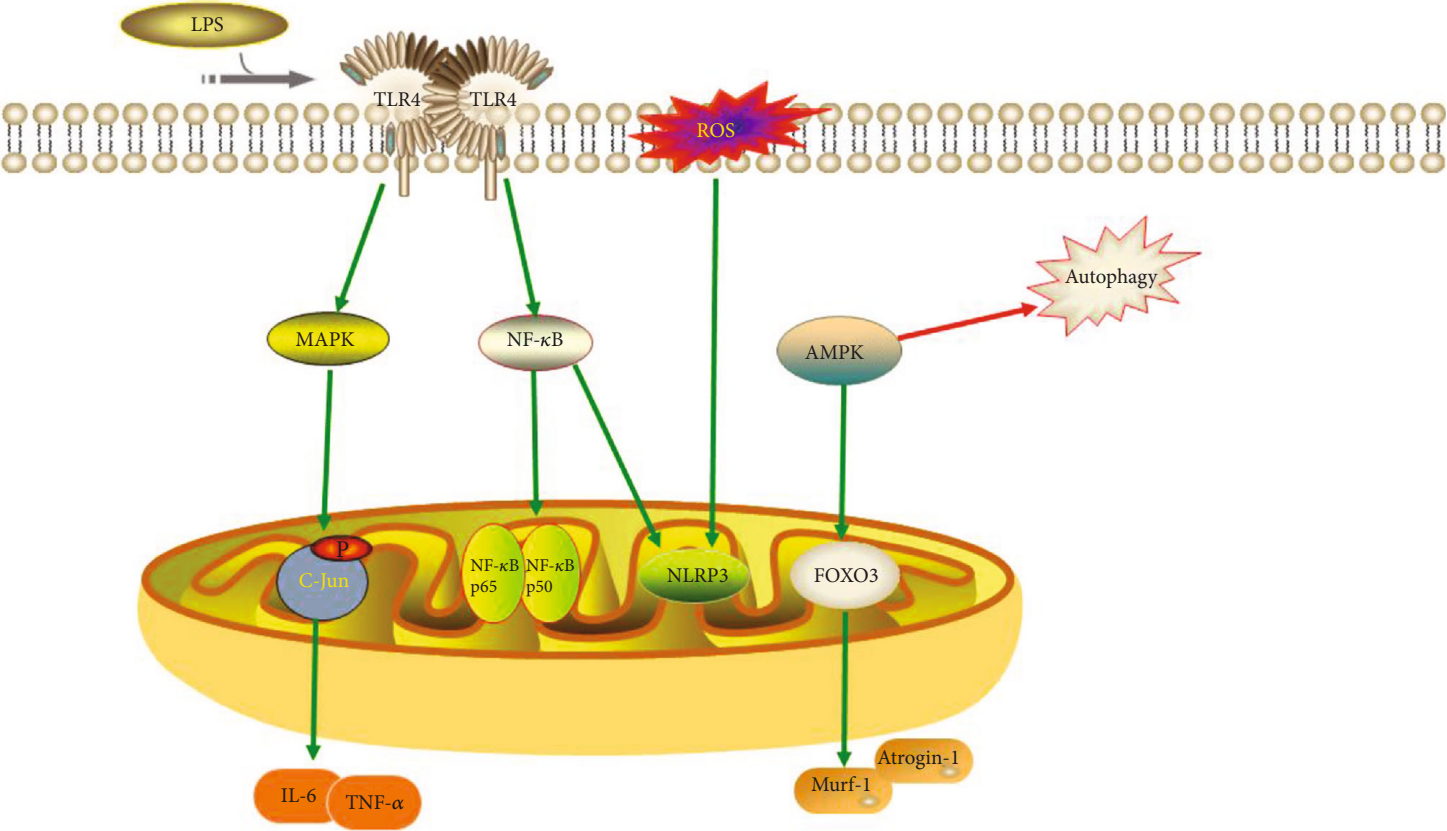
TLR4 signaling and the production of ROS induce inflammatory responses. AMPK signaling regulates autophagic activity and produces muscle atrophy factors.

One of the major mechanisms contributing to the loss of skeletal muscle mass and function is systemic chronic inflammation. As research has progressed, the importance of the gut microbiota in skeletal muscle metabolism and immunological function has become recognized. The gut microbiota promotes metabolic homeostasis and immune function by strengthening the intestinal barrier [64]. Gut microbial disorders and loss of variety, in contrast, compromise the integrity of the intestinal barrier, allowing hazardous microbial products such as lipopolysaccharide (LPS) to enter the bloodstream, and these harmful substances trigger systemic inflammation and lead to metabolic disorders and decreased muscle function and mass [15]. Elevated LPS levels activate Toll-like receptor (TLR) 4 signaling, which leads to metabolic endotoxemia [65]. Activation of the TLR4 signaling pathway causes a significant increase in nuclear factor- (NF-) *κ*B protein levels (p50 and p65) and c-Jun N-terminal kinase phosphorylation, resulting in a decrease in human immune function [65]. Specifically, the TLR4 signaling pathway induces upregulation of proinflammatory cytokines (interleukin-6 and tumor necrosis factor-*α*) through a cascade response, thereby inducing a systemic inflammatory response [66] (Figure 1).

In recent years, autophagy has received a lot of attention as a fundamental element in skeletal muscle mass and function regulation. Autophagy ensures skeletal muscle quality and function by removing dysfunctional organelles from senescent cells [67]. The AMP-activated protein kinase (AMPK) and peroxisome proliferator-activated receptor-coactivator- (PGC-) 1 signaling pathways are known to regulate cellular metabolism and play essential roles in autophagy, inflammation, insulin resistance, and skeletal muscle. In addition, AMPK and PGC-1*α* signaling pathways are associated with the gut microbiota–muscle axis [68]. The activation of AMPK and PGC-1 decreases with age [69], and inhibition of AMPK and PGC-1*α* signaling pathways decreases autophagic activity, leading to a decrease in skeletal muscle mass and function [70]. Decreased autophagic activity exacerbates the inflammatory response, which in turn inhibits activation of the AMPK signaling pathway [71]. The reduced autophagic activity also clusters dysfunctional organelles in senescent cells, thereby increasing the production of reactive oxygen species (ROS). The level of the inflammasomes, including Nod-like receptor 3 (NLRP3), is stimulated by ROS [72]. The NF-*κ*B signaling mentioned above also stimulates the production of NLRP3 inflammasomes [73]. Thus, dysregulated autophagic activity and inflammatory responses play a pivotal part in the loss of skeletal muscle mass and function, and AMPK and PGC-1*α* signaling pathways are closely associated with the gut microbiota–muscle axis [68]. Further research into the relationships between the AMPK and PGC-1 signaling pathways, autophagy, inflammatory responses, and the gut microbiome could aid in the treatment of disorders characterized by skeletal muscle mass and function loss (Figure 1).

Increased expression of atrophy marker genes, particularly Murf-1 and Atrogin-1, which play a critical role in muscle atrophy, is linked to the role of microbiota in the reduction of muscle mass and function [74]. FOXO transcription factors influence the production of Murf-1 and Atrogin-1 [74]. By activating the FOXO3-mediated protein breakdown pathway, AMPK modulates muscle fiber size [75]. Decreased muscle mass and strength in GF mice are associated with increased expression of FOXO, Murf-1, and Atrogin-1. The MyoG and FOXO3 pathways and their downstream target genes are regulated by the gut microbiota and their derived metabolites during protein synthesis and degradation [76]. The activation of AMPK signaling in GF mouse muscle suggests that the AMPK/FOXO3/Atrogin-1/Murf-1 signaling pathway may be implicated in the gut microbiota–muscle axis [23] (Figure 1).

### 3.2. Endocrine System

**Figure 2:**
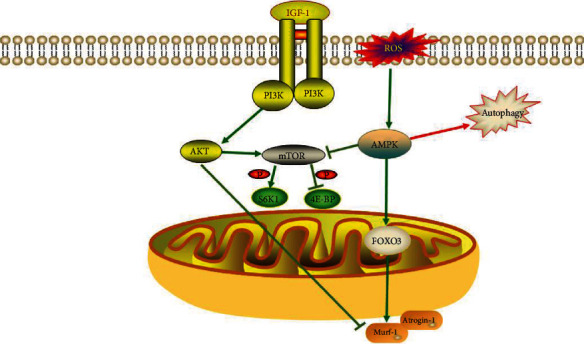
IGF-1 activates mTOR through PI3K/AKT signaling to stimulate protein synthesis. The PI3K/AKT signaling pathway inhibits the expression of myasthenic markers (Murf-1 and Atrogin-1). ROS inhibits mTOR activity by activating the AMPK signaling pathway.

The endocrine system has an important role in muscle mass regulation, with insulin, insulin-like growth factor- (IGF-) 1, and growth hormone influencing muscle growth and development [77]. In general, insulin acts on skeletal muscle to promote glucose uptake and upregulates anabolic signaling, which influences the rate of muscle protein synthesis [78]. Dysregulation of the gut microbiota and disruption of epithelial regeneration can be founded in intestinal epithelial IGF-1 gene-deficient mice compared with normal mice [79]. Mechanistically, IGF-1 regulates muscle growth through the phosphatidylinositol 3-kinase (PI3K)/Akt signaling pathway and inhibits the mRNA transcription and translation process of muscle protein synthesis (MPS) [80]. The PI3K/AKT signaling pathway is a well-known insulin-resistance pathway [81], and it is disrupted in diabetic patients. Insulin production and beta-cell activity may be diminished once this route is blocked, worsening insulin resistance even more [82]. Insulin resistance causes muscle cells to be unable to utilize glucose and instead rely on glycogen or fat, which can lead to a loss of muscle mass and function [83] (Figure 2).

**Figure 3:**
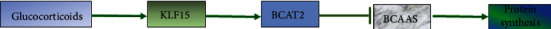
Glucocorticoids inhibit protein synthesis by activating KLF15, which leads to the degradation of BCAAs.

Glucocorticoids can induce skeletal muscle atrophy under pathological conditions [84]. One of the target genes for glucocorticoid receptor activation is Kruppel-like factor (KLF) 15, which is implicated in metabolic activities in skeletal muscle such as overexpression of branched-chain aminotransferase2, which leads to degradation of branched-chain amino acids (BCAAs) [85]. Loss of gut microbiota also leads to the degradation of BCAAs in muscle. Increased catabolism of BCAAs in GF mice is a key factor in muscle atrophy, and increased expression of genes involved in BCAA metabolism leads to reductions in muscle mass, hindlimb grip strength, and spontaneous activity in mice [23]. Catabolism of BCAAs is linked to skeletal muscle proteolysis and has the ability to modulate protein synthesis [86] (Figure 3).

### 3.3. Protein Anabolism

A balance between protein synthesis and breakdown keeps skeletal muscle mass in check. A state of negative muscle protein balance occurs when the rate of muscle protein breakdown (MPB) exceeds the rate of MPS over time, resulting in a reduction in skeletal muscle function and mass [87]. It is widely believed that the decrease in muscle function and mass is caused by diminished ability to stimulate MPS rather than by acceleration of MPB [88]; a metabolic phenomenon known as muscle anabolic resistance.

Mammalian target of rapamycin (mTOR) is a downstream target of PI3K/Akt. mTOR stimulates protein synthesis in two ways: phosphorylation and inactivation of eukaryotic initiation factor 4E-binding protein1 and phosphorylation and activation of ribosomal S6 kinase1 [89]. Many studies have demonstrated that mTOR signaling regulates MPS, and that inhibition of mTOR signaling results in decreased muscle function and muscle loss [90]. IGF-1 can activate mTOR activity by activating the PI3K/Akt signaling pathway, thereby stimulating protein synthesis [91]. Production of myasthenic markers (Murf-1 and Atrogin-1) is downregulated by the PI3K/Akt pathway [92]. However, phosphorylation and activation of AMPK can inhibit mTOR activity [93]. Decreased insulin sensitivity and inflammatory responses also reduce mTOR signaling. Reduced insulin sensitivity inhibits mTOR activity by reducing IGF-1 levels, and overproduction of inflammatory factors as well as ROS can inhibit the mTOR pathway by activating the AMPK pathway [9] (Figure 2).

**Figure 4:**
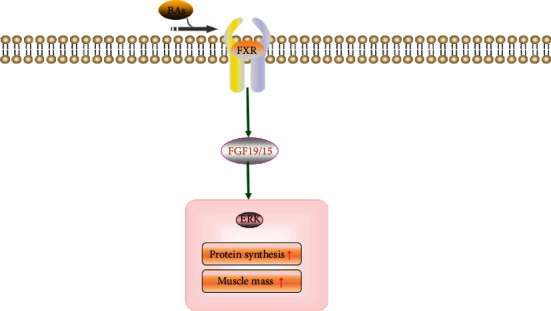
BAs promote protein synthesis and strengthen muscle mass through the FXR/FGF15/19 signaling pathway.

An increasing number of studies have shown that the gut microbiota can produce a large number of bacterial metabolites to activate diverse receptors in host cells, thus maintaining homeostasis in the host. Bile acids (BAs) are metabolites produced by the microbiota [94]. BAs bind to cellular BA receptors, one of which is the nuclear farnesoid X receptor (FXR), to modulate host glucose and lipid metabolic signaling [95]. FXR is activated in the ileum and produces fibroblast growth factor (FGF) 19, which is called FGF15 in rodents. In previous research, BAs, BA receptors, and the FXR-FGF15/19 signaling pathway have all been linked to skeletal muscle mass and function [96]. The expression of FGF15/19 activates the protein kinase (ERK) signaling pathway and phosphorylation of ERK downstream targets p90 ribosomal S6 kinase and ribosomal protein S6 to catalyze protein synthesis [97]. In short, gut microbiota disorders inhibit the BA/FXR/FGF15/19/ERK signaling pathway, resulting in restricted protein synthesis and thus skeletal muscle atrophy [98] (Figure 4).

### 3.4. Peroxisome Proliferator-Activated Receptors

Peroxisome proliferator-activated receptors (PPARs) are members of the nuclear receptor family of transcription factors that are activated by fatty acids and their derivatives. After activation by ligand binding, PPAR heterodimerizes with retinoid X receptors, forming a heterodimer that binds to a PPAR response element upstream of the target gene promoter, ultimately regulating the transcription of the target gene [99]. There are three subtypes of PPAR: PPAR*α*, *β*/*δ*, and *γ*. PPAR*α* is highly expressed not only in the liver, heart, brown adipose tissue, and kidney but also in skeletal muscle [100]. It plays an important role in fatty acid catabolism by regulating peroxisomal and mitochondrial *β*-oxidation and microsomal *ω*-oxidation of fatty acids; it is also involved in glucose metabolism and is key in controlling energy expenditure and suppressing inflammatory responses [101]. The expression of PPAR*β*/*δ* is more widespread in skeletal muscle, and it plays an important role in glucose and lipid metabolism, inflammatory response, energy expenditure, and muscle fiber type switching [102]. PPAR*γ* is highly expressed in adipocytes and is associated with lipid deposition in muscle and other organs, affecting adipogenesis as well as triglyceride storage [103].

It has been shown that mice lacking PPAR*β*/*δ* have a reduced number of muscle satellite cells with decreased regenerative capacity, ultimately leading to muscle atrophy and decreased muscle mass and body weight, suggesting that PPAR*β*/*δ* regulates postnatal myogenesis and regeneration in mice [104]. Some mice with specific active PPAR*β*/*δ* have shown greater resistance to fatigue [105]. Abnormal energy metabolism and reduced muscle fibers have been observed in mice with PPAR*β*/*δ* knockout in muscle and adipocyte hypertrophy, and glucose intolerance with insulin resistance has also been observed [106]. PGC-1*α* has been shown to be a downstream target gene of PPAR*β*/*δ* [107]. The expression of PPAR*β*/*δ* also increases the level of PGC-1*α*, which affects fatty acid oxidation and glucose metabolism [108]. These results also show that PPAR agonists can improve the deficiency of myotonic proteins, compensate for the loss of muscle fibers, and improve myotonic dystrophy [109]. Experiments using antibiotics to treat mice with changes in muscle peripheral biological clock mechanisms and metabolic regulators (PPAR*γ*) have suggested that disturbances in the gut microbiota are associated with the expression of genes that regulate muscle peripheral circadian mechanisms and metabolism [26].

PPAR primarily interacts with the gut microbiota in inflammation and metabolism [110]. PPAR*α* protects the intestine from an inflammation-induced increase in intestinal permeability by preventing neutrophil infiltration, and the microbiota activates PPAR*α* through TLR4 signaling, thereby acting to reduce inflammation [111]. Previously, it was reported that treatment of mice with type I diabetes with a PPAR*α* agonist (bezafibrate) resulted in improved skeletal muscle insulin sensitivity through activation of PI3K/AKT signaling [112]. Similarly, PPAR*β*/*δ* and PPAR*γ* play a role in reducing inflammation in the intestines, thereby regulating the composition of the intestinal flora [113]. PPAR*β*/*δ* suppresses the inflammatory response and enhances insulin sensitivity by activating the AMPK signaling pathway and inhibiting the extracellular regulated protein kinase ERK1/2 [114]. PPAR*γ* in muscle promotes glucose utilization by muscle through activation of glucose transporter protein (GLUT) 1 and GLUT4 [115].

### 3.5. Mitochondrial Function and Neuromuscular Connectivity

Skeletal muscle mitochondrial dysfunction is also a cause of decreased muscle mass and function [116]. Skeletal muscle mitochondrial function and content decrease with age, and electron microscopy shows abnormally expanded mitochondrial segments [117]. The production of IGF-1 by the gut microbiota connects mitochondrial skeletal muscle to the gut microbiota. It was discovered that IGF-1 levels in GF mice were lower than in PF mice, and that the expression of genes encoding mitochondrial oxidative phosphorylation complexes was lower in GF mouse skeletal muscle, resulting in a loss in mitochondrial function [23].

**Figure 5:**
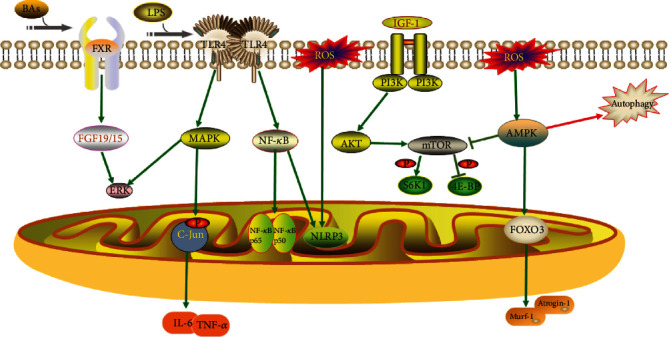
Mechanisms involved in the gut microbiota–skeletal muscle axis.

The central nervous system controls skeletal muscle function via neurotransmission at the neuromuscular junction [118]. Acetylcholine, a key neurotransmitter for signaling between muscles and nerves, was reduced in GF mice when compared to PF mice, as was the expression of the acetylcholine receptor subunit Rapsyn and low-density lipoprotein receptor-related protein 4; both of which are important for neuromuscular junction assembly [23] (Figure 5).

## 4. Interventions

**Figure 6:**
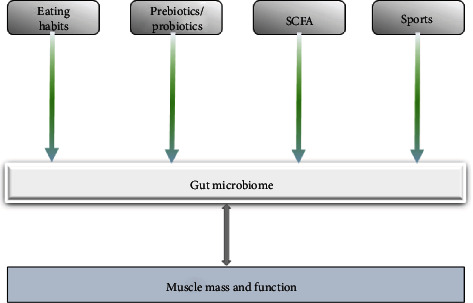
Diet, exercise, prebiotics/probiotics, and SCFA supplementation can alter the gut microbiota and improve muscle mass and function.

To date, there have been many preclinical and human studies that have directly or indirectly demonstrated a link between gut microbiota and muscle mass/function (Table 1). Various interventions have been proposed for the gut microbiota, and probiotics and/or prebiotics, SCFAs, dietary supplementation, and exercise have all been effective in enhancing muscle mass and host function (Figure 6). Dietary habits influence the composition of the gut microbiota and can induce changes in the microbiota that are important for the function of the organism [119]. In the context of skeletal muscle aging, eating disorders cause reduced microbial diversity and increased intestinal permeability, which inhibit cytokine-mediated protein anabolism [120]. Supplementation of prebiotics and/or probiotics improves intestinal homeostasis and promotes skeletal muscle metabolism and synthesis [121]. Exercise or physical activity is also a factor in regulating the gut microbiota [122].

In a mouse model, forelimb grip strength and endurance swimming time were significantly increased after 6 weeks of supplementation with *Lactobacillus plantarum* TWK10 (LP10), which increased glucose utilization and reduced the inflammatory response by increasing the number of types I muscle fibers in the gastrocnemius muscle, thereby increasing endurance exercise time [123]. In a study of *Drosophila*, *Lactobacillus plantarum* can increase protein synthesis and upregulate mTOR, thereby promoting MPS and enhancing muscle anabolism [124]. Curcumin as a prebiotic can alter the composition of gut microbiota and improve endurance, swimming time, and forelimb grip strength in mice, possibly due to a significant increase in tissue glycogen content in mice after supplementation with nanobubble curcumin extract (NCE) [125]. Inulin combined with microbial transplantation improves endurance in mice on a low microbiome-accessible carbohydrate (LMC) diet, but no improvement in muscle mass was found [126]. Myotube diameter was significantly reduced after treatment of mouse skeletal muscle C2C12 myotubes with dexamethasone, whereas *Lactobacillus curvatus* CP2998 (CP2998) restored mouse myotube diameter by inhibiting glucocorticoid receptor activation and prevented muscle atrophy [127]. After oral administration of kefir supplementation, the forelimb grip strength scores, endurance swimming time, and muscle mass of mice were significantly higher than in controls, and the composition of the gut microbiota of mice was changed (reduced *Firmicutes/Bacteroidetes* ratio) and tissue glycogen content was also significantly increased after kefir supplementation [128]. After oral administration of *Lactobacillus casei* LC122 or *Bifidobacterium longum* BL986 for 12 weeks, these two probiotics improved intestinal barrier function, increased muscle strength, and reduced oxidative stress and inflammation in peripheral tissues [129]. *Lactobacillus paracasei* PS23 restores mitochondrial dysfunction due to aging in mice, reduces inflammatory factor activity, and has potential therapeutic implications for decreased skeletal muscle function and quality [130]. Colonization of *Eubacterium rectale* or *Clostridium coccoides* in mice increases endurance swimming fatigue time [131]. *Veillonella atypica* was isolated from fecal samples of marathon runners. Inoculation of this strain into mice significantly increases treadmill running exhaustion time, and *Veillonella atypica* improves running time by converting exercise-induced lactate metabolism to propionic acid [132]. Transferring fecal samples from older adults (high-functioning/low-functioning group) into GF mice found significantly increased grip strength in high-functioning mice compared to low-functioning mice [52]. Treatment with *Faecalibacterium prausnitzii* increased muscle mass in high-fat-fed mice, which may be associated with enhanced mitochondrial respiration, altered intestinal microbiota composition, reduced inflammatory response, and improved intestinal integrity [133]. *Lactobacillus salivarius subspecies salicinius* (SA-03) was isolated from the gut microbiota of gold medal weight lifters and then orally fed to mice for 4 weeks, resulting in a significant improvement in muscle strength and endurance performance and an increase in liver and muscle glycogen stores [134]. Similarly, *Bifidobacterium longum* (OLP-01), isolated from gold medal winners in weightlifting, was supplemented into mice and found that OLP-01 supplementation improved grip strength and endurance in mice and significantly increased liver and muscle glycogen levels [135]. Compared with GF mice, mice in the *Bacteroides fragilis* group showed increased endurance swimming time, reduced physical fatigue, and lower serum superoxide dismutase activity than GF mice [54].

Many studies have demonstrated that the gut microbiota can produce SCFA by fermenting indigestible carbohydrates [136]. SCFAs consist of three primary components: acetate, propionate, and butyrate; all of which are absorbed in the intestinal lumen and influence muscle and fat metabolism [137]. After feeding SCFA to GF mice, it was found that GF mice showed greater gastrocnemius muscle mass and strength, and the grip strength of GF mice was increased, which was consistent with the fact that SCFA increased muscle density, muscle mass, and function in GF mice by regulating the expression of Atrogin-1 and Murf-1 [23]. Butyrate prevents the loss of skeletal muscle mass and function during aging. After butyrate treatment, aged mice were found to have increased muscle fibers, prevented intramuscular fat accumulation, decreased fat mass in mice, and improved glucose metabolism and mitochondrial function in skeletal muscle [138].

After 13 weeks of oral administration of prebiotics consisting of a mixture of inulin plus fructooligosaccharides to elderly people aged 65 and over with frailty syndrome, these participants were found to have improved muscle strength and reduced fatigue, possibly because the prebiotics affected the body's immune function by promoting the growth of beneficial bacteria, inhibiting the growth of pathogens, and reducing other proinflammatory cytokines [139]. In triathletes, *Lactobacillus plantarum* PS128 increased endurance running performance, which was linked to changes in microbiota composition and greater levels of SCFAs [140]. *Lactobacillus plantarum* TWK10 has been shown in previous studies to improve exercise performance in mouse models, and LP10 has also been shown to do the same in human experiments. In healthy adults taking LP10 daily, it was found that LP10 significantly increased human exercise capacity in a dose-dependent manner, as well as improved fatigue-related performance and significantly increased muscle mass [141]. An observational study of older men found that a diet high in dietary fiber had higher physical performance indicators, higher scores on the short physical performance battery (SPPB), and higher grip strength, and that a diet high in dietary fiber may have a positive effect on the body's production of butyrate [142]. In a test of 32 sedentary older women over the age of 65, 12 weeks of aerobic training altered the participants' gut microbiota diversity and increased trunk muscle strength, and fecal SCFA level content has also been increased [143]. After supplementing 10 male runners with probiotic capsules daily for 4 weeks, it was found that probiotic supplements significantly increased runners' fatigued exercise time in the heat [144]. In a test of young adult female swimmers, it was found that after 8 weeks of supplementation with probiotic yogurt, the athletes' aerobic performance improved [145].

## 5. Conclusion and Future Perspectives

**Figure 7:**
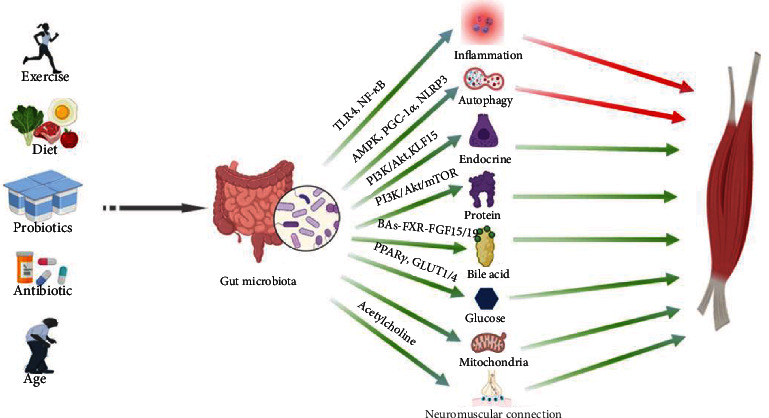
The gut–muscle axis under physiological and pathological conditions. Red arrows represent negative effects on muscles, and green arrows represent positive effects on muscles.

The role of the gut microbiota–muscle axis plays a crucial role in both humans and animals. The gut microbiota interacts with skeletal muscle through inflammatory immunity, autophagy, protein anabolism, energy, lipids, neuromuscular connectivity, oxidative stress, mitochondrial function, and endocrine and insulin resistance, thus affecting the physiological functions of the body (Figure 7). Specifically, the host's diet provides nutritional resupply to the gut microbiota, which maintains the structural integrity and the health of the gut, and participates in and mediates nutrient absorption and metabolism in the gut, which provides the material basis for muscle growth and development. Substances such as neurotransmitters, SCFAs, and bile acids produced by the metabolism of the gut microbiota regulate energy consumption and storage through the nervous and circulatory systems, providing energy for muscle development. The gut microbiota also influences the secretion of insulin, glucocorticoids, and leptin through the endocrine system, hormones that are important regulators of muscle growth and development. In addition, disturbance of the gut microbiota and invasion of exogenous harmful substances can lead to the impaired intestinal barrier and increased secretion of proinflammatory cytokines, which can negatively affect muscle growth and development.

Dietary supplementation, probiotics and/or prebiotics, SCFAs, and exercise can influence the composition of the gut microbiota, improving skeletal muscle mass and function. Although there is now a large body of research demonstrating a strong link and communication between gut microbiota and muscle tissue, there are no clear experiments showing which type or types of probiotics and/or prebiotics, SCFA, promote muscle growth and development, and there is also a lack of research on the quantitative nature of supplements.

To validate the above influencing factors and the mechanisms involved, a large number of high-quality interventional experimental studies are needed to demonstrate how dietary supplementation, probiotics and/or prebiotics, SCFAs, and exercise affect the gut microbiota. It is believed that as research methods continue to advance, the understanding of the gut microbiota–muscle axis will become more advanced. By regulating the gut microbiota, people can improve several diseases caused by reduced skeletal muscle mass and function.

## Figures and Tables

**Table 1:** The effects of gut microbiota on skeletal muscle.

References	Objects	Methods	Results	Remarks
Chen et al. [123]	Mice	Supplementation of LP10	Forelimb grip strength and endurance swimming time were increased	LP10 reduces the inflammatory response, improves glucose utilization, and increases the number of type I muscle fibers in the gastrocnemius muscle
Storelli et al. [124]	Drosophila	Supplementation of *Lactobacillus plantarum*	Increased protein synthesis and enhanced muscle anabolism	Upregulation of mTOR pathway and enhancement of MPS
Chen et al. [125]	Mice	Supplementation of NCE	Forelimb grip strength and endurance swimming time were increased	NCE alters gut microbiota composition and increases tissue glycogen content
Okamoto et al. [126]	LMC diet mice	Inulin supplementation combined with microbial transplantation	Endurance was improved	Muscle mass improvement was not found, and it may be difficult to promote muscle growth with a single supplement of inulin
Katsuki et al. [127]	Mice	Supplementation of *Lactobacillus curvatus* CP2998	The myotubular diameter was restored	CP2998 prevents dexamethasone-induced muscle atrophy by inhibiting glucocorticoid receptor activation
Hsu et al. [128]	Mice	Supplementation of kefir	Significant improvement in forelimb grip strength score, endurance swim time, and muscle mass	Altered gut microbiota composition and increased tissue glycogen content
Ni et al. [129]	Mice	Supplementation of *Lactobacillus casei* LC122 or *Bifidobacterium longum* BL986	Improved muscle strength and function	Improved intestinal barrier function and reduced inflammatory response
Chen et al. [130]	Mice	Supplementation of *Lactobacillus paracasei* PS23	Reduced risk of sarcopenia	Improved mitochondrial function and decreased secretion of proinflammatory cytokines
Huang et al. [131]	Mice	Colonization of *Eubacterium rectale* or *Clostridium coccoides*	Endurance swimming time was increased	/
Scheiman et al. [132]	Mice	Inoculation of *Veillonella atypica*	Treadmill running exhaustion time was increased	*Veillonella atypica* converts lactic acid metabolism to propionic acid
Fielding et al. [52]	Mice	Fecal samples from older adults	The grip strength of mice in the high-function group increased significantly	Altered gut microbiome and strengthened intestinal barrier in high-functioning mice
Munukka et al. [133]	Mice	Supplementation of *Faecalibacterium prausnitzii*	Muscle mass was increased	Enhanced mitochondrial respiration, reduced inflammatory response, altered gut microbiota composition, and improved intestinal integrity
Lee et al. [134]	Mice	Supplementation of SA-03	Significant improvement in muscle strength and endurance performance	Increased liver and muscle glycogen stores, decreased levels of lactate, blood urea nitrogen, ammonia, and creatine kinase
Lee et al. [135]	Mice	Supplementation of OLP-01	Increased grip strength and endurance in mice	Increased SCFA, liver, and muscle glycogen
Hsu et al. [54]	Mice	Supplementation of *Bacteroides fragilis*	Increased muscle mass and endurance swimming time	Serum superoxide dismutase activity was lower than GF mice
Lahiri et al. [23]	Germ-free mice	Supplementation of SCFA	Increased muscle mass and function and grip strength	SCFA reduces the expression of Atrogin-1 and Murf-1
Walsh et al. [138]	Mice	Supplementation of butyrate	Prevention of hind limb muscle atrophy in mice	Increase in muscle fibers, prevention of intramuscular fat accumulation, improvement of mitochondrial function and glucose metabolism
Buihues et al. [139]	Elderly people (≥65 years)	Supplementation of prebiotic:inulin plus fructooligosaccharides	Improved muscle strength and endurance, less fatigue	Prebiotics promote the growth of beneficial bacteria and reduce proinflammatory cytokines
Huang et al. [140]	Triathletes	Supplementation of *Lactobacillus plantarum* PS128	Significantly improves triathletes' endurance	Regulate gut microbiota composition and increase SCFA content
Huang et al. [141]	Healthy adults	Supplementation of LP10	Increased muscle mass and fatigue resistance	LP10 improves aerobic endurance performance
Barger et al. [142]	Older men	High dietary fiber diet	Higher grip strength and physical performance indicators	High dietary fiber promotes butyrate production
Morita et al. [143]	Older women	12 weeks of aerobic training	Increased trunk muscle strength	Increased gut microbiota diversity and fecal SCFA content
Shing et al. [144]	Male runners	Supplementation of probiotic capsules	Prolonged fatigue exercise at high temperatures	/
Salarkia et al. [145]	Female swimmers	Supplementation of probiotic yogurt	Improved aerobic performance	Improved maximum oxygen uptake
